# Fermentation to Increase the Value of Roasted Coffee Silverskin as a Functional Food Ingredient

**DOI:** 10.3390/foods14152608

**Published:** 2025-07-25

**Authors:** Nadia Guzińska, Maria Dolores del Castillo, Edyta Kordialik-Bogacka

**Affiliations:** 1 Interdisciplinary Doctoral School, Lodz University of Technology, 116 Stefana Żeromskiego Street Lodz, 90-543 Lodz, Poland; nadia.guzinska@dokt.p.lodz.pl; 2 Institute of Fermentation Technology and Microbiology, Faculty of Biotechnology and Food Sciences, Lodz University of Technology, 171/173 Wólczańska, 90-530 Lodz, Poland; 3 Instituto de Investigación en Ciencias de la Alimentación (CIAL) (CSIC-UAM), Campus de la Universidad Autónoma de Madrid, C/Nicolás Cabrera, 9, 28049 Madrid, Spain

**Keywords:** roasted coffee silverskin, microbial fermentation, antioxidant capacity, phenolic compounds, probiotic viability, symbiotic microbial culture consortium, functional food ingredients, prebiotic potential, clean label, zero waste

## Abstract

Roasted coffee silverskin (RCSS) is a by-product of coffee production characterized by its content of phenolic compounds, both free and bound to macromolecules. In this study, RCSS was fermented to release these compounds and consequently increase its value as a functional food ingredient. Fermentation was carried out using yeast, acetic acid bacteria, and lactic acid bacteria, either as single strains or as a designed microbial consortium. The latter included *Saccharomycodes ludwigii*, *Gluconobacter oxydans*, and *Levilactobacillus brevis*, mimicking a symbiotic culture of bacteria and yeast commonly used in kombucha fermentation (SCOBY). This symbiotic microbial culture consortium demonstrated notable efficacy, significantly enhancing the total phenolic content in RCSS, with values reaching 14.15 mg GAE/g as determined by the Folin–Ciocalteu assay and 7.12 mg GAE/g according to the Fast Blue BB method. Antioxidant capacity improved by approximately 28% (ABTS) and 20% (DPPH). Moreover, the fermented RCSS supported the viability of probiotic strains (*Saccharomyces boulardii* SB01 and *Levilactobacillus brevis* ŁOCK 1152) under simulated intestinal conditions. These results suggest that RCSS, particularly after fermentation with a full symbiotic microbial culture consortium, has strong potential as a clean label, zero-waste functional food ingredient.

## 1. Introduction

The concepts of clean label (CL) and zero waste are increasingly intertwined, driving the use of by-products from food production in the creation of new, natural food products [1,2]. The term “clean label” gained prominence in the early 2010s as an extension of the natural ingredient trend [3]. CL has become a key segment of the food market, especially appealing to consumers who prefer products with a simple composition made from a limited number of easily recognizable ingredients [4]. The growing awareness of production methods and food composition has also contributed to the rising interest in the “zero waste” movement. This concept focuses on reducing waste, both at the production and consumption levels [5]. Zero-waste initiatives have gained popularity alongside increased environmental awareness and concern about climate change. Guided by the concepts of CL and zero waste, the food industry is striving to develop innovative solutions that meet consumer demand for transparency, sustainability, and environmental responsibility [6].

Coffee is one of the most widely used raw materials, and its production generates significant amounts of waste [7]. Some by-products of coffee production, such as cascara and spent coffee grounds [8,9], have already been introduced successfully to the food market. Roasted coffee silverskin (RCSS) is a by-product of the coffee roasting process. Coffee silverskin (CSS) is a thin tegument that directly covers the coffee seed, which becomes detached during roasting [10]. CSS accounts for 4.2% of the total seed weight, resulting in the production of 1 ton of RCSS for every 120 tons of roasted coffee [11]. The management of this waste represents a significant challenge for all coffee-producing nations, underscoring the critical importance of identifying innovative applications for this by-product within the framework of sustainable development.

The phenolic composition of RCSS is expected to closely resemble that of the roasted coffee beans from which it is derived and includes compounds such as chlorogenic, caffeic, and coumaric acids [12]. Other chemical components of RCSS include dietary fiber (50–60%), protein (16–19%), fat (1.56–3.28%), and ash (7%) [13]. Due to roasting, a significant proportion of phenolic compounds in RCSS becomes bound to complex structures, including melanoidins, dietary fiber, and protein matrices, reducing their extractability and biological activity. Microbial fermentation is a promising strategy with which to release these bound compounds through enzymatic activity (e.g., β-glucosidase, esterases, and proteases), thereby enhancing the functional potential of RCSS [14].

Furthermore, the microbial metabolism of dietary fiber can generate short-chain fatty acids (SCFAs) and other fermentation products with prebiotic effects [15]. Therefore, both the liberated phenolic compounds and microbial metabolites derived from fiber fermentation may contribute to modulating gut microbiota and promoting intestinal health. This dual contribution reinforces the relevance of fermented RCSS as a clean label, antioxidant, and prebiotic ingredient for functional food applications [16,17].

Due to the high stability of RCSS resulting from its low moisture content, it is a promising additive for food products [18]. Recent studies have shown that RCSS can be successfully incorporated into products such as yogurt, cookies, and hamburger patties [19,20,21]. RCSS has also gained attention for its potential prebiotic properties due to its high fiber content and the presence of bioactive compounds. Rich in polysaccharides and phenolic compounds, RCSS can stimulate the growth of beneficial intestinal microbiota, potentially enhancing gut health and metabolic function when incorporated into functional foods. However, RCSS is considered a novel food in the EU, as it was not consumed in significant quantities as food before 15 May 1997. Consequently, it requires pre-market authorization under Regulation (EU) 2015/2283 before it can be marketed as food within the EU [22].

Fermentation is one of the key processes enhancing both the sensory qualities and functional properties of food products [23]. It has been used for centuries to increase the bioavailability of bioactive compounds, including phenols [24], and represents a powerful tool for transforming underutilized by-products such as RCSS into high-value functional ingredients. In this context, microbial consortia such as the symbiotic culture of bacteria and yeast (SCOBY), commonly used in kombucha, offer synergistic metabolic interactions among yeasts, acetic acid bacteria (AAB), and lactic acid bacteria (LAB), enhancing substrate utilization and producing functional metabolites [25,26]. While previous studies have shown the feasibility of fermenting coffee by-products such as cascara and spent grounds using SCOBY [27,28], no prior research has evaluated the fermentation of RCSS for functional food applications.

The aim of this study was to investigate the effects of RCSS fermentation on its phenolic content, antioxidant activity, and prebiotic potential, indicated by the improved viability of selected probiotic strains (*Saccharomyces boulardii* SB01 and *Levilactobacillus brevis* ŁOCK 1152) under simulated intestinal conditions. The selected strains, *Saccharomycodes ludwigii*, *Gluconobacter oxydans*, and *Levilactobacillus brevis*, were chosen for their relevant metabolic traits. These include acid and polyphenol tolerance, β-glucosidase activity, antioxidant capacity, and potential to produce bioactive compounds with prebiotic effects [29,30,31,32,33,34,35,36,37,38,39,40]. Prebiotics are defined as “a non-nutritive food ingredient that provides health benefits to the host associated with modulation of the microbiota” [41], and play an increasingly important role in functional food design [42].

## 2. Materials and Methods

### 2.1. Materials

#### 2.1.1. Roasted Silver Skin of Coffee Beans

**Figure 1:**
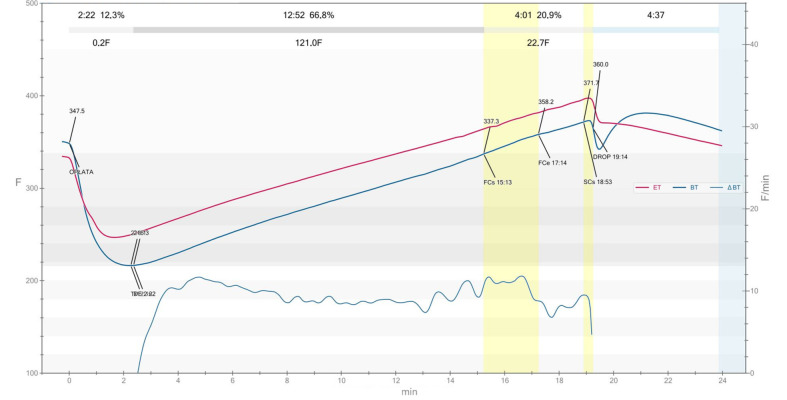
The roasting curve of the process by which RCSS used in the study was obtained. The designation ET stands for environment temperature, while BT refers to bean temperature. The point marked as “DROP” on the graph indicates the moment when roasting is completed by removing the beans from the roaster.

The RCSS obtained by roasting Columbia Supremo (*C. arabica* L.) beans were provided by the coffee roastery Cafe Roma (Piotrków Trybunalski, Poland). Figure 1 illustrates a coffee roasting profile where the bean temperature (BT) increases steadily from approximately 175.3 °C at first crack start (FCs) to 198.3 °C at second crack start (SCs), with the first crack end (FCe) at 195.2 °C at 16:13 min. The drying phase lasted 2:22 min (12.3%), the Maillard phase 12:52 min (66.8%), and the development time (post-first crack) 4:01 min (20.9%), indicating a medium roast level. The rate of rise (RoR) curve shows a stable decline during the majority of the roast. Overall, the profile reflects a controlled roast aimed at promoting sweetness and flavor complexity. The roasting process curve was generated using Artisan v3.2.0 software (Artisan-Software LLC, Omaha, NE, USA). The bromatological characteristic of the raw material is present in Table S1 as Supplementary Material. The study was conducted 7 days after the roasting process was complete.

#### 2.1.2. Microorganisms

The yeast *S. ludwigii* WSL/A3 (Y), acetic acid bacteria *G. oxydans* ŁOCK 1153 (AAB), and lactic acid bacteria *L. brevis* ŁOCK 1152 (LAB) were used in the study. All of the microorganisms were sourced from the Collection of Pure Microbial Cultures ŁOCK (Łódź, Poland). The strains were selected based on observed growth on RCSS in previously conducted experimental assessments. The microorganisms Y, AAB, and LAB were used for fermenting RCSS either individually or in specific configurations.

The inoculums were cultured in 10 mL of the appropriate medium: yeast on Yeast Peptone Glucose (YPG), acetic acid bacteria on Glucose-Free Yeast Peptone (GFYP), and lactic acid bacteria on De Man, Rogosa, and Sharpe (MRS) broth (Merc) at 30 °C for 48 h.

### 2.2. Methods

#### 2.2.1. Food Safety of RCSS

The microbial quality of the RCSS was evaluated following the method described by Osimani [43], with modifications. To estimate viable cells in RCSS, 1 g of each sample was aseptically diluted in 9 mL of sterile distilled water. Ten-fold dilutions of the homogenates were then inoculated on suitable solid media for the counting of various microbial groups. Total mesophilic aerobes were grown in standard Plate Count Agar (PCA) (Merc) at 30 °C for 48 h. Aerobic bacterial spores were cultivated in standard PCA at 30 °C for 48 h, after thermal treatment of the homogenates at 80 °C for 15 min followed by cooling in iced water for 5 min. The lactic acid bacteria were cultured on MRS agar supplemented with nystatin (60 mg/L) at 37 °C for 48 h. *Enterobacteriaceae* were grown on Violet Red Bile Glucose Agar (VRBGA) (Merc) at 37 °C for 24 h. *Eumycetes* were cultured at 25 °C for 72 h on Sabouraud Dextrose Agar (SDA) (Merc) supplemented with chloramphenicol (100 mg/L).

#### 2.2.2. Fermentation

For fermentation, 5 g of RCSS was suspended in 45 mL of sterile distilled water and inoculated with 5.5 log CFU/mL of the chosen strain. When a consortium of strains was used, each strain was inoculated at a concentration of 5.5 log CFU/mL. The fermentation process was carried out at 30 °C for 48 h.

#### 2.2.3. Number of Viable Microbial Cells in RCSS During Fermentation

The numbers of viable microbial cells in the RCSS were quantified using the plate count method after 0, 24, and 48 h of fermentation. For this purpose, 1 g of fermented RCSS was aseptically diluted in 9 mL of sterile distilled water. Ten-fold dilutions of the homogenates were then inoculated on suitable solid media. The yeasts were cultivated on SDA (Merc) supplemented with chloramphenicol (100 mg/L) at 30 °C for 72 h. The acetic acid bacteria were grown on Orange Serum Agar (OSA) (Merc) supplemented with nisin (60 mg/L), nystatin (60 mg/L), and 10% of 96% ethanol (*v*/*v*) at 30 °C for 72 h. The lactic acid bacteria were cultured on MRS agar (Merc) supplemented with nystatin (60 mg/L) at 37 °C for 48 h. The results are presented as the logarithm (log) of colony-forming units (CFU) per gram.

#### 2.2.4. Measurements of pH

The pH of RCSS samples was measured after 0, 24, and 48 h of fermentation using a calibrated pH meter (Elmetron CP-411, Elmetron, Zabrze, Poland). To reduce the risk of microbial contamination of the fermentation regime, a portion of each sample was aseptically collected and designated specifically for pH determination. The pH was measured directly by immersing the electrode into the biomass, ensuring accurate and representative readings.

#### 2.2.5. Overall Antioxidant Capacity and Total Phenolic Compounds

To assess antioxidant capacity, 5 g of the RCSS was extracted using 50 mL of 80% methanol. After 30 min, the mixture was centrifuged at 6000 rpm for 20 min. The antioxidant capacity of the RCSS before fermentation and after 48 h of fermentation was measured using the DPPH assay described by Herald [44] and the ABTS assay described by Iriondo-DeHond [45]. The TPC in the unfermented RCSS and after 48 h of fermentation were determined using the Folin–Ciocalteu (FC) method described by Ainsworth [46] and the Fast Blue BB (FBBB) method described by Medina [47]. Gallic acid was used as a standard substance. The results were recorded as mg of gallic acid (GAE) per gram of the sample.

#### 2.2.6. Prebiotic Properties

The prebiotic properties of unfermented and fermented RCSS were determined by measuring the viability of probiotic yeast *S. boulardii* and bacterium *L. brevis* following exposure to simulated intestinal fluid (SIF) supplemented with RCSS according to the adapted procedure described by Siroli et al. [48]. A higher number of microorganisms (log CFU/mL) throughout the incubation period in the SIF supplemented with RCSS in comparison to SIF alone indicated greater resistance of probiotic microorganisms to gastrointestinal conditions, and consequently prebiotic properties of RCSS.

The probiotic yeast strain *S. boulardii* SB01 was isolated from a commercially available probiotic formulation and plated on SDA (Merck KGaA, Darmstadt, Germany) supplemented with chloramphenicol (100 mg/L) to inhibit bacterial growth. The lactic acid bacteria *L. brevis* ŁOCK 1152 from the Pure Culture Collection of Industrial Microorganisms (ŁOCK 105) at the Institute of Fermentation Technology and Microbiology, Lodz University of Technology (Poland), was cultivated on MRS agar (Merck) supplemented with nystatin (60 mg/L) to suppress fungal contaminants. Both cultures were incubated at 36 °C for 24 h. For the simulated intestinal fluid (SIF: 0.1% *w/v* pancreatin (Sigma-Aldrich, St. Louis, MO, USA), 0.15% *w/v* Oxgall bile salt (Carl Roth GmbH + Co. KG, Karlsruhe, Germany), pH 7), *S. boulardii* SB01 or *L. brevis* ŁOCK 1152 was inoculated at a final concentration of approximately 5 log CFU/mL into SIF containing 0.1 g of the tested unfermented and fermented RCSS. Samples were collected after 0, 6, 12, and 24 h of exposure to SIF and plated on the corresponding selective media (SDA or MRS). Incubation was carried out at 37 °C for 24 h under anaerobic conditions, using the GasPak EZ Anaerobic System (Becton, Dickinson and Co., Sparks, MD, USA).

#### 2.2.7. Statistical Analysis

Statistical analysis was carried out using STATGRAPHICS 18 software (Manugistics Inc. Rockville, MD, USA). Statistical significance was assessed using a one-way ANOVA with a 95% confidence level.

## 3. Results and Discussion

### 3.1. Food Safety of RCSS

Microbiological analysis of the RCSS indicated no microbial contamination and food safety (Table 1). Low numbers of mesophilic aerobic bacteria, measured at only 0.2 ± 0.1 (log CFU/g), were detected. No bacterial spores, lactic acid bacteria, *Enterobacteriaceae* or *Eumycetes* were detected. The absence of *Enterobacteriaceae*, a large family of bacteria that includes potential pathogens such as *E. coli*, is important, and implies high microbiological purity [49]. These results align with findings reported by Nolasco [50], who also observed that high temperatures during the roasting process and the low moisture content of RCSS create unfavorable conditions for the growth of most microorganisms. The results confirm the absence of contamination by the tested pathogens associated with foodborne illnesses, ensuring the safety of the product for consumption.

### 3.2. Changes in pH Values Due to RCSS Fermentation

The pH of the unfermented RCSS ranged from 4.90 to 4.92. These values are lower than those reported by Martuscelli [18] and Hashimoto [51]. The lower pH obtained in this study may result from the use of RCSS from different coffee species. A low pH in RCSS is advantageous and is probably attributed to the presence of chlorogenic, citric, tartaric, ferulic, and malic acids [52]. The pH value decreased significantly over time during fermentation, with both single strains and microbial consortia (Table 2). During fermentation, beneficial acids such as lactic, gluconic, and acetic acid may be produced by the microorganisms used. Gluconic acid imparts a refreshing sour taste in many beverages, such as wine and fruit juices [53]. In addition, valuable coffee-derived polyphenols, such as caffeic acid and chlorogenic acid, enter the ferment as a result of the degradation of lignin, protein–phenol complex, polysaccharide–phenol complex, and melanoidins [14]. A significant reduction in pH was observed after both 24 h and 48 h. The highest decrease was noted during fermentation with the symbiotic microbial culture consortium of yeast, acetic acid bacteria, and lactic acid bacteria, while the lowest was observed for fermentations with yeast or LAB. This indicates that fermentation with AAB and with consortia of two microorganisms (sample 5: Y + AAB; sample 6: Y + LAB; and sample 7: LAB + AAB) can achieve a similar reduction in pH. Currently, no literature data are available for direct comparison with these findings.

### 3.3. RCSS as a Medium for the Microorganisms’ Growth

#### 3.3.1. Growth of Microorganisms During RCSS Fermentation

In this study, a significant increase in the number of microbial cells was noted during the first 24 h of fermentation. A decrease in the cell numbers was observed in the subsequent 24 h for all fermentation processes (Table 3). The growth of bacteria was more intense than the growth of yeast, regardless of whether the bacteria were applied as single strains or in consortia. Lactic acid bacteria reached the highest concentrations, peaking at 7.9 ± 0.10 (log CFU/g) during 24 h fermentation with the full consortium. The highest growth of yeast was also observed during 24 h fermentation with the full consortium, but yeast growth reached only 5.7 ± 0.11 (log CFU/g). The results of this study suggest that RCSS has a similar effect on the growth of AAB and LAB. However, the number of AAB was lower than the number of LAB, with a difference of 0.26 ± 0.21 (log CFU/g).

RCSS can be a valuable medium for microbiomes due to its high fiber and nitrogen content [54]. However, there are only a few reports on the use of RCSS as a substrate for microbial growth [55,56]. Polysaccharides and melanoidins, Maillard reaction products generated during coffee roasting, composing RCSS are fermentable macromolecules [57]. Melanoidins would either inhibit the growth of pathogenic microorganisms or support the growth and metabolites of probiotic ones [14,58]. On the other hand, RCSS contains lignin, which limits the availability of fermentable compounds. Phenols, alkaloids, and tannins have an inhibitory effect on the growth of fermenting microorganisms [59].

The limited growth of yeast may be attributed to potential fermentation inhibitors, including furfural, 5-HMF, caffeine, and antioxidants [60,61]. These compounds are found in lignocellulosic biomass including RCSS and may have a more pronounced effect on yeast metabolism than on the metabolism of bacteria. The impact of inhibitory substances is limited when microorganisms are in a consortium. It has been shown that LAB degrade caffeine, one of the potential fermentation inhibitors, and contribute to improving most quality parameters [62].

Concurrently, the release of greater amounts of inhibitory compounds over time could have inhibited the growth of microorganisms and reduced their numbers on the second day of fermentation. However, this effect might also be partially attributed to the depletion of fermentable macromolecules, which are essential for microbial metabolism. Interestingly, although phenolic compounds are often considered inhibitory, some have been reported to exhibit prebiotic properties, potentially supporting the growth of beneficial bacteria under certain conditions. Supplementation of the growth medium with RCSS to enhance LAB growth might therefore be a cost-effective and time-efficient solution to improving the cultivation of beneficial bacteria in applications such as fermented foods [53,63].

#### 3.3.2. Survival of Probiotic Microorganisms Exposed to SIF Containing RCSS

**Figure 2:**
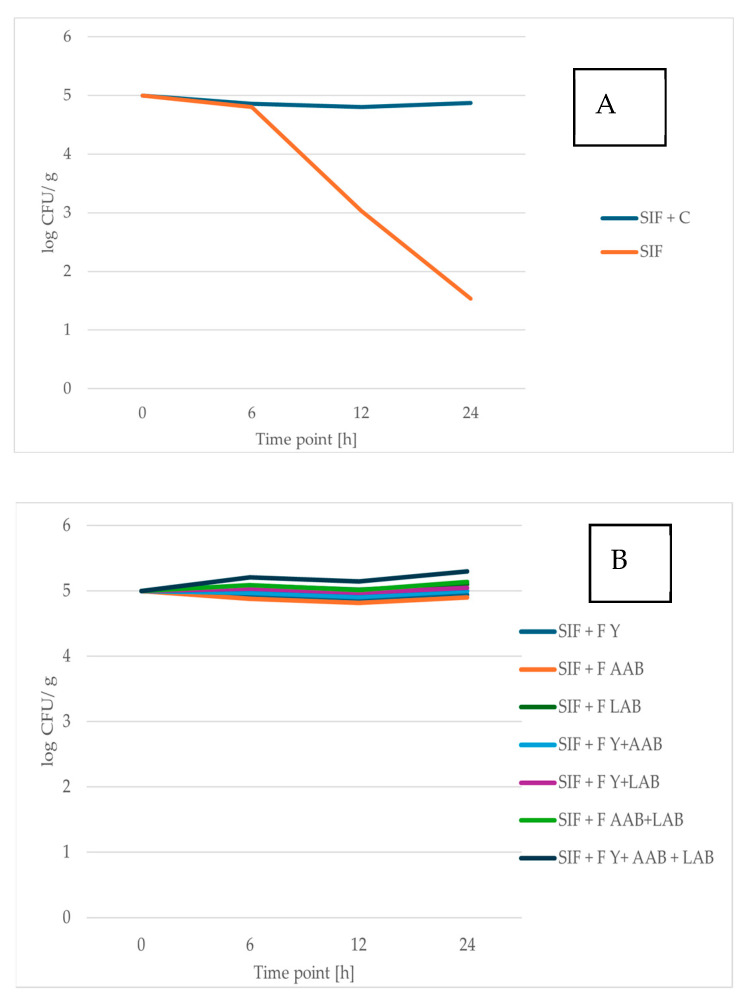
Viability of *L. brevis* ŁOCK 1152 after 0, 6, 12, and 24 h of exposure to simulated intestinal fluid (SIF). (**A**) SIF containing raw roasted coffee silverskin (RCSS) and SIF alone (control). (**B**) SIF containing fermented RCSS. Results are shown as mean ± standard deviation (n = 3).

**Figure 3:**
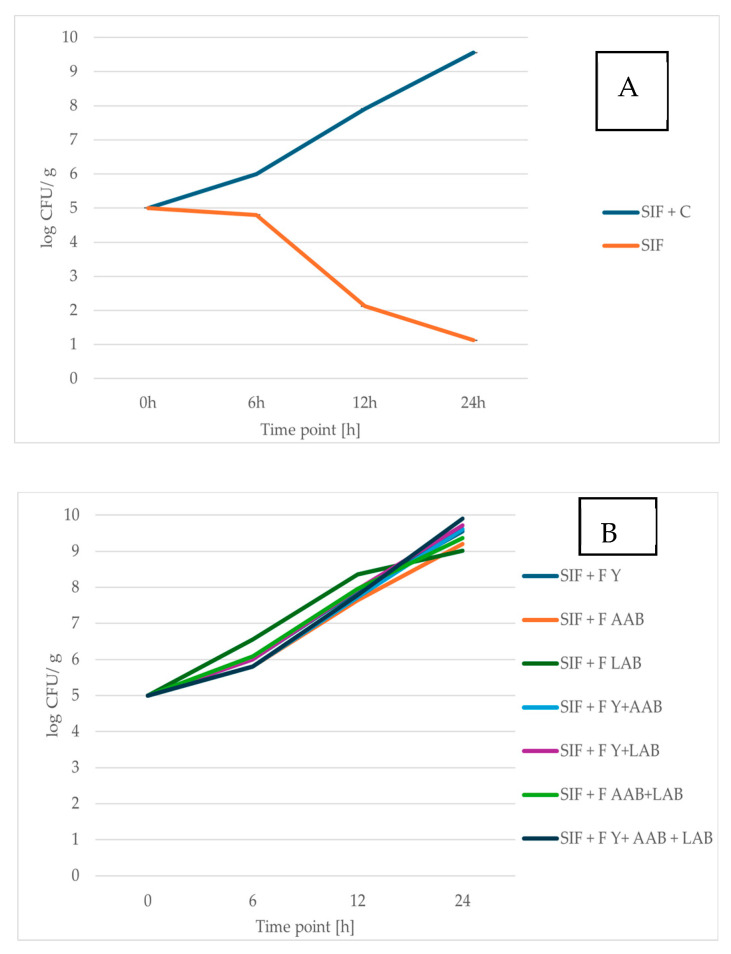
Viability of *S. boulardii* SB01 after 0, 6, 12, and 24 h of exposure to simulated intestinal fluid (SIF). (**A**) SIF containing raw roasted coffee silverskin (RCSS) and SIF alone (control). (**B**) SIF containing fermented RCSS. Results are shown as mean ± standard deviation (*n* = 3).

Prebiotic foods are attracting growing interest from consumers due to their important role in maintaining a healthy balance of gut microorganisms and promoting overall well-being [64]. This study supports previous findings indicating that RCSS influences the growth of probiotic bacteria positively [51,59] (Figure 2). In addition, RCSS also stimulated the proliferation of probiotic yeast strain (Figure 3). Comprehensive data and associated statistical analyses are presented in Table S1 of the Supplementary Material.

The prebiotic properties of unfermented and fermented RCSS were evaluated by assessing the viability of the probiotic yeast *S. boulardii* and the bacterium *L. brevis* following exposure to SIF supplemented with RCSS.

When incubated in SIF alone, both probiotic yeast and bacterial strains exhibited reduced viability. After 24 h, the number of viable *S. boulardii* cells declined by 77.56% of the initial population, while the viable count of *L. brevis* decreased by 69.45%. However, when RCSS was added to the SIF, the survival of both probiotic strains significantly improved. After 24 h in RCSS-supplemented SIF, the viable cell count of *L. brevis* was 3.29-fold higher, and that of *S. boulardii* was 8.42-fold higher compared to SIF alone. The probiotic yeast, *S. boulardii,* demonstrated a more pronounced increase in viability than the bacterial strain, reaching over 4 log CFU/mL after 24 h. Owing to its high resilience and well-documented health benefits, *S. boulardii* represents a promising candidate for the development of probiotic food products [30,65].

The *L. brevis* strain also showed strong viability in RCSS-supplemented SIF, with its population remaining stable throughout the 24 h incubation. These findings suggest that RCSS plays an important role in stabilizing probiotic microorganisms under gastrointestinal-like conditions. This stabilizing effect of RCSS supplementation can be compared to microencapsulation, a commonly applied method for enhancing the viability of probiotic strains [66].

The viability of the single strain in SIF decreases notably after 6 h of incubation. While the RCSS formulation supports the survival of the tested strains, the symbiotic microbial consortium F Y + AAB + LAB demonstrates the most pronounced protective effect, resulting in the highest viable cell count. Fermentation processes altered the chemical composition of RCSS, enhancing the availability of certain growth-promoting compounds. Notably, supplementation with RCSS fermented by Y + LAB or Y + LAB + AAB led to significantly higher counts of *S. boulardii* after 24 h of incubation. The greatest increase in the number of *L. brevis* cells was observed in SIF enriched with RCSS fermented using the consortium of Y + AAB + LAB.

Overall, these results highlight the potential of both fermented and unfermented RCSS as a valuable material with prebiotic effects for application in the food industry.

### 3.4. Total Phenolic Compounds and Overall Antioxidant Capacity

Fermentation with single strains of AAB, LAB, and yeast, as well as with microbial consortia, can enhance total phenolic content in a variety of food products [67]. Coffee and its by-product, cascara, have also been successfully subjected to fermentation processes [68,69]. However, to date, fermentation has not been investigated as a strategy for valorizing RCSS. Additionally, this study is the first to apply the FBBB method to determine phenolic compounds in RCSS, as no previous reports in the literature have used this technique for this matrix. Importantly, the FBBB assay is more selective for phenolic compounds than the widely used FC method, which can also react with non-phenolic reducing agents such as proteins, amino acids, and sugars [70,71]. This greater specificity explains why the TPC values obtained using the FBBB method were consistently lower than those from the FC assay. In this study, the TPC in fermented RCSS varied depending on the microorganisms used (Table 4).

Fermentation with monocultures did not result in statistically significant changes in phenolic compound concentration. The lowest TPC measured by the FC method (8.25 ± 0.04 mg GAE/g) was observed in the sample fermented with AAB. The FBBB assay showed that TPC values for monoculture-fermented samples ranged from 4.02 ± 0.13 to 4.19 ± 0.04 mg GAE/g, which did not significantly differ from the control sample (3.41 ± 0.02 mg GAE/g). This may be attributed to the narrow metabolic activity of monocultures, which limits the range of biochemical transformations and can even result in the degradation of certain phenolic compounds [72,73]. In contrast, fermentation with microbial consortia led to a significant increase in TPC. The highest TPC value measured by the FC method (14.15 ± 0.06 mg GAE/g) was observed in the sample fermented with the symbiotic microbial culture consortium composed by yeast, AAB, and LAB, representing a 32% increase compared to the control. This result was further confirmed by the FBBB method, which recorded 7.12 ± 0.31 mg GAE/g for the same sample, indicating an important increase over the control. These findings highlight the benefits of microbial synergy, where interactions among different strains enhance metabolic diversity and lead to a greater accumulation of phenolic compounds [74]. To assess the antioxidant potential of fermented RCSS, both DPPH and ABTS assays were employed. Fermentation positively influenced antioxidant capacity, although no statistically significant differences were observed between monocultures and consortia. Melanoidin fractions present in RCSS have the capacity to produce SCFEs and release antioxidants, thereby modulating microbial activity [14]. The highest radical scavenging activity was recorded in RCSS fermented with the full microbial consortium: 27.69 ± 0.86% inhibition using the ABTS assay and 20.43 ± 0.33% using the DPPH assay. Fermentation with LAB and yeast consortia notably enhanced phenolic content and antioxidant properties. A relationship was observed between TPC and antioxidant activity, along with a decrease in pH during fermentation. A mean correlation (R^2^ = 0.751) was observed between TPC, represented by FC and FBBB, and antioxidant activity, measured by DPPH and ABTS assays, in the fermented samples. This suggests that phenolic acids produced or liberated during fermentation contribute to the acidification of the medium, further influencing the metabolic profile of fermented RCSS and promoting phenolic compound accumulation.

## 4. Conclusions

RCSS, the by-product of the coffee roasting process, represents a promising material for functional food applications due to its high content of phenolic compounds and fermentable molecules. This study demonstrated that the fermentation of RCSS using selected microbial strains, *S. ludwigii, G. oxydans,* and *L. brevis,* named the symbiotic microbial culture consortium, can effectively enhance its total phenolic content and overall antioxidant capacity.

Fermentation not only improved the bioactive profile of RCSS, but also increased its ability to support the survival of probiotic strains, including *S. boulardii* and *L. brevis*, under simulated intestinal conditions. The most pronounced effects were observed when using a symbiotic microbial culture consortium consisting of yeast, acetic acid bacteria, and lactic acid bacteria, highlighting the synergistic interactions between these microorganisms.

The findings indicate that both fermented and unfermented RCSS may serve as a valuable substrate with prebiotic properties, contributing to the viability and stability of probiotic microorganisms. Incorporating RCSS into functional food formulations aligns with CL and zero-waste principles, offering a sustainable strategy for the valorization of coffee industry by-products.

This study explores a novel approach for the valorization of RCSS through fermentation and encourages further research to advance the scientific understanding and practical applications of fermented RCSS. Future investigations should focus on in vivo assessments of its effects on gut microbiota composition, immune modulation, and metabolic health parameters. Additionally, studies assessing the sensory attributes and functional properties of food products incorporating fermented RCSS into real food matrices are highly recommended.

## Figures and Tables

**Table 1:** Microbial contamination of RSCC.

Type of Microorganisms	Number of Cells [log CFU/g]
Mesophilic aerobic bacteria	0.2 ± 0.10
Lactic acid bacteria	n.d.
*Enterobacteriaceae*	n.d.
Bacterial spores	n.d.
*Eumycetes*	n.d.

The abbreviation n.d. stands for “not detected”.

**Table 2:** Values of pH in three time points of RCSS fermentation (0, 24, and 48 h).

Sample	0 h	24 h	48 h
C	4.9 ± 0.00 ^Aa^	4.89 ± 0.00 ^Ca^	4.89 ± 0.00 ^Ca^
F Y	4.91 ± 0.00 ^Ac^	4.45 ± 0.00 ^Bb^	4.36 ± 0.00 ^Ba^
F AAB	4.91 ± 0.00 ^Ac^	4.40 ± 0.00 ^ABb^	4.33 ± 0.00 ^ABa^
F LAB	4.90 ± 0.00 ^Ac^	4.45 ± 0.00 ^Bb^	4.36 ± 0.00 ^Ba^
F Y + AAB	4.91 ± 0.00 ^Ac^	4.41 ± 0.00 ^ABb^	4.32 ± 0.00 ^ABa^
F Y + LAB	4.91 ± 0.00 ^Ac^	4.39 ± 0.00 ^ABb^	4.30 ± 0.00 ^ABa^
F AAB + LAB	4.91 ± 0.00 ^Ac^	4.4 ± 0.00 ^ABb^	4.32 ± 0.00 ^ABa^
F Y + AAB + LAB	4.92 ± 0.00 ^Ac^	4.38 ± 0.00 ^Ab^	4.28 ± 0.00 ^Aa^

Results are expressed as mean ± standard deviation (*n* = 3). Different letters in each row indicate significant differences (*p* < 0.05) according to one-way ANOVA. Uppercase letters (A–C) indicate differences between samples at the same time point; lowercase letters (a–c) indicate differences across time points for the same sample. Samples: C = unfermented RCSS (control); F Y = fermented with *S. ludwigii*; F AAB = with *G. oxydans*; F LAB = with *L. brevis*; F Y + AAB = with *S. ludwigii* + *G. oxydans*; F Y + LAB = with *S. ludwigii* + *L. brevis*; F AAB + LAB = with *G. oxydans* + *L. brevis*; and F Y + AAB + LAB = with all three strains, also called symbiotic microbial culture consortium.

**Table 3:** Effect of fermentation conditions, RCSS presence, inoculum used, and fermentation time on the growth of microorganisms (log CFU/g).

Microbial Strain	Time Point [h]	C	F Y	F AAB	F LAB	F Y + AAB	F Y + LAB	F AAB + LAB	F Y + AAB + LAB
*S. ludwigii*	0	n.d.	5.5 ± 0.10 ^Aa^	n.d.	n.d.	5.54 ± 0.03 ^Aa^	5.52 ± 0.21 ^Aa^	n.d.	5.49 ± 0.14 ^Aa^
	24	n.d.	5.69 ± 0.08 ^Ac^	n.d.	n.d.	5.69 ± 0.23 ^Ac^	5.69 ± 0.07 ^Ac^	n.d.	5.7 ± 0.91 ^Ac^
	48	n.d.	5.64 ± 0.56 ^Ab^	n.d.	n.d.	5.68 ± 0.08 ^Bb^	5.66 ± 0.13 ^Bb^	n.d.	5.66 ± 0.02 ^Bb^
*G. oxydans*	0	n.d.	n.d.	5.53 ± 0.45 ^Aa^	n.d.	5.52 ± 0.34 ^Aa^	n.d.	5.52 ± 0.54 ^Aa^	5.51 ± 0.21 ^Aa^
	24	n.d.	n.d.	7.34 ± 0.13 ^Ac^	n.d.	7.31 ± 0.19 ^Bc^	n.d.	7.61 ± 0.21 ^Cc^	7.64 ± 0.63 ^Cc^
	48	n.d.	n.d.	7.23 ± 0.26 ^Ab^	n.d.	7.27 ± 0.42 ^Bb^	n.d.	7.58 ± 0.21 ^Db^	7.61 ± 0.06 ^Cb^
*L. brevis*	0	n.d.	n.d.	n.d.	5.53 ± 0.18 ^Aa^	n.d.	5.52 ± 0.15 ^Aa^	5.5 ± 0.06 ^Aa^	5.51 ± 0.02 ^Aa^
	24	n.d.	n.d.	n.d.	7.68 ± 0.18 ^Ac^	n.d.	7.89 ± 0.21 ^Ac^	7.90 ± 0.14 ^Bc^	7.90 ± 0.16 ^Bc^
	48	n.d.	n.d.	n.d.	7.63 ± 0.03 ^Ab^	n.d.	7.82 ± 0.21 ^Ab^	7.87 ± 0.02 ^Bb^	7.83 ± 0.04 ^Bb^

Results are expressed as mean ± standard deviation (*n* = 3). Fermentations were conducted at 0, 24, and 48 h using eight conditions: C = unfermented RCSS (control); F = fermented RCSS with Y = *S. ludwigii*, AAB = *G. oxydans*, LAB = *L. brevis*, or their combinations. Different uppercase letters within the same row indicate significant differences (*p* < 0.05) due to the microbial strain or combination used, and different lowercase letters within the same column indicate significant differences (*p* < 0.05) due to the fermentation time for the specific microorganism being analyzed. The abbreviation n.d. stands for “not detected”.

**Table 4:** Total phenolic content and antioxidant activity of RCSS after 48 h fermentation process with various microorganisms.

Sample	FC	FBBB	DPPH	ABTS
	(GAE mg/g)		Inhibition (%)	
C	10.72 ± 0.01 ^AB^	3.41 ± 0.02 ^A^	10.03 ± 0.19 ^A^	21.48 ± 1.28 ^A^
F Y	9.29 ± 0.04 ^A^	4.19 ± 0.04 ^AB^	17.68 ± 2.87 ^B^	25.36 ± 1.86 ^B^
F AAB	8.25 ± 0.04 ^A^	4.12 ± 0.01 ^AB^	18.04 ± 3.15 ^B^	25.47 ± 2.31 ^B^
F LAB	8.70 ± 0.06 ^A^	4.02 ± 0.13 ^AB^	18.34 ± 2.12 ^B^	25.23 ± 1.06 ^B^
F Y + AAB	12.28 ± 0.09 ^BC^	4.15 ± 0.18 ^AB^	19.25 ± 0.29 ^B^	23.60 ± 1.16 ^B^
F Y + LAB	14.09 ± 0.13 ^C^	3.81 ± 0.11 ^AB^	19.59 ± 0.55 ^B^	25.84 ± 1.79 ^B^
F AAB + LAB	13.94 ± 0.11 ^C^	4.30 ± 0.06 ^B^	20.35 ± 3.44 ^B^	23.64 ± 3.96 ^B^
F Y + AAB + LAB	14.15 ± 0.06 ^C^	7.12 ± 0.31 ^C^	20.43 ± 0.33 ^B^	27. 69 ± 0.86 ^B^

Results are expressed as mean ± standard deviation (n = 3). Fermentations were conducted at 0, 24, and 48 h using eight conditions: C = unfermented RCSS (control); F = fermented RCSS with Y = *S. ludwigii*, AAB = *G. oxydans*, LAB = *L. brevis*, or their combinations. Different uppercase letters within the same column indicate significant differences (*p* < 0.05) due to the microbial strain or combination used, and different lowercase letters within the same column indicate significant differences (*p* < 0.05) due to the fermentation time for the specific microorganism being analyzed.

## Data Availability

The original contributions presented in the study are included in the article/Supplementary Material, further inquiries can be directed to the corresponding authors.

## Supplementary Materials

The following supporting information can be downloaded at: https://www.mdpi.com/article/10.3390/foods14152608/s1 [75,76], Table S1: Content of components in RCSS; Table S2: Viability of *S. boulardii* SB01 and *L. brevis* ŁOCK 1152 after 0, 6, 12, 24 h of exposure to SIF containing unfermented and fermented RCSS.

## Abbreviations

The following abbreviations are used in this manuscript:

RCSS	Roasted Coffee Silver Skin
CSS	Coffee Silver Skin
SCOBY	Symbiotic Cultures of Bacteria and Yeast
CL	Clean Label
ROS	Reactive Oxygen Species
Y	The yeast S. ludwigii WSL/A3
AAB	The acetic acid bacteria G. oxydans ŁOCK 1153
LAB	The lactic acid bacteria L. brevis ŁOCK 1152
YPG	Yeast Peptone Glucose Medium
GFYP	Glucose-Free Yeast Peptone Medium
MRS	De Man, Rogosa, and Sharpe Medium
PCA	Plate Count Agar
VRBGA	Violet Red Bile Glucose Agar Medium
SDA	Sabouraud Dextrose Agar Medium
OSA	Orange Serum Agar
CFU	Colony-Forming Units
GAE	Gallic Acid
SIF	Simulated Intestinal Fluid
FC	Folin–Ciocalteu
FBBB	Fast Blue BB
