# Supplementary material for: Fermentation to Increase the Value of Roasted Coffee Silverskin as a Functional Food Ingredient
Source: Foods. 2025 Jul 25;14(15):2608. doi: 10.3390/foods14152608 (PMC12346125; doi:10.3390/foods14152608)
Supplement: Supplementary file 1 [file foods-14-02608-s001.zip › foods-3724924-supplementary.pdf]

Fermentation to increase the value of roasted coffee silverskin as a functional food ingredient

Nadia Guzińska [1, 2,*], Maria Dolores del Castillo [3*], Edyta Kordialik-Bogacka [2*]

[1] Interdisciplinary Doctoral School, Lodz University of Technology, 116 Stefana Żeromskiego Street Lodz, 90-543 Lodz, Poland
[2] Institute of Fermentation Technology and Microbiology, Faculty of Biotechnology and Food Sciences, Lodz University of Technology, 171/173 Wólczańska, 90-530 Lodz, Poland.
[3] Instituto de Investigación en Ciencias de la Alimentación (CIAL) (CSIC-UAM), C/ Nicolás Cabrera, 9,Campus de la Universidad Autónoma de Madrid, 28049 Madrid, Spain.

*nadia.guzinska@dokt.p.lodz.pl (NG), mdolores.delcastillo@csic.es (MDC), edyta.kordialik-bogacka@p.lodz.pl (EKB)

1. Bromatological Characterization of RCSS

In order to better characterize the raw material used, its composition was determined through bromatological analyses. The methods used for the determination of ash, moisture, protein, and fiber were based on standard procedures [75], while fat content was determined according to Gottstein [76]. Carbohydrates were calculated via difference, i.e., by subtracting the sum of moisture, ash, protein, fat, and fiber from 100%, which is a widely accepted approach in compositional analysis.

Table S1. Content of components in RCSS.

Component	Content [%]
Ash	7.83 ± 0.08
Moisture	1.89 ± 0.98
Protein	13.13 ± 0.75
Fat	3.35 ± 0.24
Carbohydrates	5.56 ± 0.30
Fiber	68,24 ± 0,65

Results are shown as mean ± standard deviation (n = 3).

2. Survival of probiotic microorganisms exposed to SIF containing RCSS

Table S2. Viability of *S. boulardii* SB01 and *L. brevis* ŁOCK 1152 after 0, 6, 12, 24 h of exposure to SIF containing unfermented and fermented RCSS.

Microbial strain	Time point [h]	SIF + C	SIF + F Y	SIF + F AAB	SIF + F LAB	SIF + F Y+AAB	SIF + F Y+LAB	SIF + F AAB+LAB	SIF + F Y+ AAB + LAB	SIF
S. ludwigii	0	5.02±0.07^{A}	5.12±0.14^{A}	5.09±0.02^{A}	5.03±0.14^{A}	5.06±0.12^{A}	5.03±0.05^{A}	5.03±0.05^{A}	5.05±0.06^{A}	5.02±0.13^{A}
	6	6.00±0.07^{C}	6.00±0.12^{C}	5.81±0.02^{B}	6.56±0.04^{E}	5.81±0.00^{B}	6.00±0.08^{C}	6.08±0.09^{D}	5.81±0.01^{B}	4.80±0.02^{A}
	12	7.91±0.09^{E}	7.91±0.04^{E}	7.65±0.06^{B}	8.36±0.09^{H}	7.73±0.02^{C}	7.94±0.08^{F}	7.96±0.02^{G}	7.79±0.08^{D}	2.12±0.03^{A}
	24	9.56±0.02^{E}	9.56±0.07^{E}	9.20±0.06^{C}	9.01±0.09^{B}	9.61±0.06^{F}	9.71±0.03^{G}	9.36±0.05^{D}	9.91±0.02^{H}	1.12±0.09^{A}
L. brevis	0	5.02±0.01^{A}	5.02±0.02^{A}	5.07±0.15^{A}	5.03±0.15^{A}	5.03±0.20^{A}	5.03±0.06^{A}	5.02±0.02^{A}	5.06±0.11^{A}	5.03±0.09^{A}
	6	4.86±0.09^{B}	4.91±0.06^{D}	4.88±0.04^{C}	5.08±0.17^{G}	4.97±0.06^{E}	5.03±0.09^{F}	5.09±0.02^{H}	5.21±0.02^{I}	4.80±0.02^{A}
	12	4.80±0.12^{B}	4.84±0.12^{D}	4.82±0.08^{C}	5.02±0.03^{G}	4.91±0.08^{E}	4.97±0.12^{F}	5.01±0.04^{H}	5.15±0.01^{I}	3.03±0.02^{A}
	24	4.87±0.23^{B}	4.94±0.05^{D}	4.90±0.01^{C}	5.10±0.05^{G}	5.00±0.15^{E}	5.04±0.02^{F}	5.14±0.02^{H}	5.30±0.00^{I}	1.53±0.04^{A}

Results are shown as mean ± standard deviation (n = 3). Different letters in each row indicate significant difference ($P < 0.05$) according to one-way ANOVA test and refers to differences between different samples at the same time point.
